# Mental Rotation as an Indicator of Motor Representation in Patients with Mild Cognitive Impairment

**DOI:** 10.3389/fnagi.2015.00238

**Published:** 2015-12-23

**Authors:** Julien Bourrelier, Alexandre Kubicki, Olivier Rouaud, Lionel Crognier, France Mourey

**Affiliations:** ^1^Institut National de la Santé et de la Recherche Médicale-U1093, Faculté des Sciences du SportDijon, France; ^2^Université Bourgogne Franche-Comté, Faculté des Sciences du SportDijon, France; ^3^Resource and Research Memory Center, University Hospital of DijonDijon, France

**Keywords:** motor imagery, aging, mild cognitive impairment, hand judgment laterality task, Alzheimer's disease

## Abstract

This internal representation of movement of part(s) of the body is involved during Implicit Motor Imagery tasks (IMI); the same representations are employed in the laterality judgment task. Few studies have looked at the consequences of aging, Alzheimer's disease (AD) and mild cognitive impairment (MCI) on the processes of motor preparation but none showed evidence of an alteration of action representation in patient with amnestic MCI. In the present study, the IMI task was used to assess the action representation abilities in MCI patients and healthy counterparts. A total of 24 elderly participants aged between 65 and 90 years old (12 women, 73.4 ± 6 years, mean ± S.D.) were recruited: 12 patients with MCI (MCI group) and 12 healthy aged adults (HAA group). The results showed that MCI patients have significantly a greater response time (RT) than HAA subjects only in IMI task and more precisely when performing their mental rotation at the challenging conditions. Furthermore, the IMI task related to the non-dominant hand induced a significant increase of RT only in MCI subjects. At the light of these results, we assume that MCI patients are able to engage themselves in IMI processes, still showing a compelling impairment of this mental ability across its complexity.

## Introduction

The aging can to be accompanied of cognitive impairments when could reach the pathological state. Alzheimer's disease (AD) research has focused on the neuropsychological features of neurodegenerative dementia such as memory deficits, aphasia, apraxia, and agnosia. According to NINCDS-ADRDA criteria, these impairments significantly disturb patients' autonomy in their everyday life activities (McKhann et al., 1984, 2011; Dubois et al., 2007). Mild cognitive impairment (MCI) with memory complaints is described as a stage at which sufferers have a high risk of developing AD in the coming years. It is characterized by a greater cognitive decline than expected in aging without a significant disruption in one's daily functionality (DeCarli, 2003; Chertkow et al., 2007; Albert et al., 2011). The earliest symptoms are abnormal amnesia and a decline of attentional control of executive functions (Perry and Hodges, 1999; Sperling et al., 2011; Simon et al., 2012). Interestingly, several authors highlighted the onset and gradual increase in motor impairments throughout the course of the illness (Scarmeas et al., 2004; Buchman and Bennett, 2011). These motor alterations in MCI and AD patients mainly concern certain features of gait and balance function, and lead to an increased risk of falling (Van Iersel et al., 2004; Camicioli et al., 2006) they also impair the realization of fine movements (Yan et al., 2008). The realization of action requires several biomechanical abilities to execute the movement, but also several neuronal processes to plan, to program and to control this action (Harris and Wolpert, 1998; Wolpert and Ghahramani, 2000).

The motor mechanism associated with the motor preparation of action follows the common neurophysiological pathway according to simulation theory (Jeannerod, 2001). These processes, involving motor imagery, allow us to create a mental simulation of movement without concomitant execution. This internal representation of movement of part(s) of the body is involved during Implicit Motor Imagery tasks (IMI) (Jeannerod, 1994; Decety, 1996); the same representations are employed in the laterality judgment task (Parsons, 1987, 1994). The participant mentally manipulates the hand stimulus to determine whether the stimulus is a left or right hand. During IMI tasks, participants imagine moving their own hands into the orientation and the view of the stimulus to determine the laterality. If the participant is engaged in an embodied mental process, the duration of the mental rotation task is linked to the stimuli orientations, which reflect different biomechanical constraints (Parsons, 1994; Decety, 1996; Thayer and Johnson, 2006). The IMI shares the same biomechanical and temporal properties with the physical execution of rotation movement (Decety et al., 1989; Sirigu et al., 1995; Papaxanthis et al., 2003). From a neurophysiological point of view, this IMI task engages the cortical and subcortical motor systems involved in motor planning and execution of action with the motor and premotor areas, the posterior parietal cortex, the basal ganglia and the cerebellum (Alivisatos and Petrides, 1997; Ganis et al., 2000; Vingerhoets et al., 2001).

Normal aging disturbs motor representation abilities. Saimpont and colleagues showed a decrease in performance in aged healthy subjects compared with young subjects through an increase in response time and error rate on the IMI task (Saimpont et al., 2009). Few studies have looked at the consequences of AD and MCI on motor preparation processes. Indeed, the literature on this subject reports impaired motor-planning processes in most AD patients and slightly impaired processes in MCI patients (Ghilardi et al., 1999; Manckoundia et al., 2006), as well as a deficit in transforming the visual input into motor output (Tippett and Sergio, 2006) and the deterioration of motor inhibition during imitation tasks (Bisio et al., 2012).

In the present study, the IMI task was used to assess action representation abilities in patients with amnestic MCI. Different levels of task difficulty were used in order to highlight the potential decline in their motor representation. If MCI is found to have an impact on motor imagery ability, greater interest should be paid to preventive strategies that target motor abilities in aged people with cognitive impairment.

## Materials and methods

### Patients and control subjects

A total of 24 elderly participants aged between 65 and 90 years old (12 women, mean age 73.4 ± 6 years) participated in this experiment. This study was carried out in accordance with the recommendations of local ethics guidelines, the Local Ethic Committee of Burgundy hospital centers (Dijon University Hospital—CHU-CMRR-France) with written informed consent from all subjects. All subjects gave written informed consent in accordance with the Declaration of Helsinki. The protocol was approved by the Local Ethic Committee and this study was assimilated in routine care, under the monitoring of the neurologist in memory center. The participants were distributed into two groups, 12 patients with MCI associated with AD (memory complaints; MCI group) and 12 healthy aged adults (HAA group). For each participant, we collected age, gender, and education level (see Table 1). All of the participants were right handed according to the Edinburgh handedness inventory (Oldfield, 1971) and had normal or corrected vision. The healthy volunteers were confirmed as non-demented according to standardized dementia tests and the mini mental state examination (MMSE) (Folstein et al., 1975), which roughly evaluates the cognitive abilities of subjects. A score above 28 out of 30 define normal cognition, while scores below this threshold show mildly, moderately or severely impaired cognitive abilities.

**Table 1:** **Demographics and RT performance in different tasks in both groups (means and SDs)**.

	**MCI (*n* = 12)**	**HAA (*n* = 12)**
Age (years)	75 ± 5.9	71.1 ± 6.5
Gender (Male)	4(8)	8(4)
Education (years)	12.00 ± 2.8	13.33 ± 1.4
MMSE^**^	23.8 ± 2.7	29.8 ± 0.4
SRT (ms)	541.91 ± 83.1	493.13 ± 75.1
CRT (ms)	769.30 ± 104.4	725.93 ± 104.7
IMI (ms)^**^	2341.81 ± 348.6	1701.6 ± 226.1

*Mini mental State Examination (MMSE) evaluates the cognitive abilities of aged subjects*.

** *Significant group difference p < 0.001*.

The diagnosis of MCI with probable AD was based on NINCDS-ADRDA criteria (McKhann et al., 1984, 2011; Albert et al., 2011) and was made by a neurologist and a specialized medical team in the national Center for Memory Resource and Research (CMRR of Dijon University Hospital, France). Patients were selected and included on the basis of neurological, neuropsychological and neuro-imaging examinations and following patients' consent. For the MCI group, the inclusion criteria were an MMSE score between 15 and 27. Below a score 15, the cognitive abilities are so severely impaired that it would be impossible for patients to understand instructions associated with tasks in the study. MCI subjects presented no pathological deficits in the test to assess apraxia, in visual-constructive abilities, which alone would explain impairment in IMI tasks (mean score of Rey's figure test, 34.4/36 ± 2.3 with a cut-off of 28/36, which is the threshold for normal abilities). Finally, for both groups, we included participants who obtained 70% or more correct responses in the first series of mainly IMI tasks.

### Materials

In the IMI tasks, the stimuli used were realistic representations of right and left hands in black and white provided by Poser software. We used the left and right hands, in back and palm views and with 8 orientation angles from 0° to 315° in steps of 45°. These stimuli were presented in a random order through software created by the computer engineer of the laboratory and projected through a screen (19″, 482.6 mm). Two response pedals were placed below the right and left feet. The right pedal was pushed as quickly as possible by the right foot to give the response “right hand” and the left pedal was pushed as quickly as possible by the left foot to give the response “left hand.” All participants were seated in front of a screen with their hands placed on their thighs. The subjects had to maintain the posture with the palms of their hands facing upwards throughout the experiment. Indeed, if participants moved their arms or hands to give the response, the change in their own posture and the movement could affect decision making notably through sensory, visual and proprioceptive feedback (Ionta and Blanke, 2009).

The IMI session consisted of 4 blocks of 32 stimuli (2 hands × 2 views × 8 orientation angles). Each hand stimulus was repeated one time per block, which started with a fixation cross, displayed for a variable interval (1.5–3 s). The stimulus remained displayed until the answer was given. When a response was provided, the message “Response recorded” was displayed and the experimenter had to validate the recording to continue the series with the next fixation cross. The confirmation of the experimenter was necessary to ensure that the attention of subject was focused on the next task and not dissipated.

In addition, each participant performed two control tasks: a simple reaction time (SRT) was measured in response to a visual stimuli (a white circle with a diameter of 6 cm) on the screen and a choice reaction time task (CRT) was used to evaluate the duration of treatment to differentiate between two control stimuli that were not matched to a body part: either an arrow pointing to the right or an arrow pointing to the left. The same stimuli presentation protocol and the same response paradigm were used: the participant had to push as quickly as possible on the right pedal in the SRT and on the right or left pedals in the CRT depending on the direction of the arrow. Each subject performed two blocks of SRT and CRT: one at the beginning and another in the middle of the experiment. Altogether, each participant performed 128 IMI trials, 20 SRT trials, and 36 CRT trials, leading to a total experiment time of 40 min.

### Data analysis

For each condition, we recorded the responses time (RT) and the error rate. The RT in the three tasks was defined as the time between the presentation of a stimulus and the moment the response is given. RT exceeding two standard deviations for all the tasks was excluded from the RT analysis. Only correct answers were taken into account for RT analysis. The error rate was defined as the proportion of wrong answers for each condition. The performance was considered above chance level when the proportion of wrong answers was below 33% for the IMI task (50/128) and below 25% for the control task (according to a binomial test, *p* < 0.001).

All dependent variables were analyzed according to the influence of factors inherent to the different tasks (SRT, CRT, and IMI task). The homogeneity of variances and normality of variables were checked beforehand by the Levene tests and the Shapiro-Wilk test, respectively. Specifically, for the IMI task, the factors HAND (left and right), VIEW (back and palm), and ORIENTATION (8 levels: from 0 to 315°in steps of 45°) for two groups (HAA and MCI) were analyzed by means of a repeated measures ANOVA. *Post-hoc* analyses were carried out using LSD Fisher tests. The alpha-level was set at *p* = 0.05.

## Results

The participants in the HAA and MCI groups did not differ in terms of age and education (see Table 1). The difference between the two groups for MMSE scores (*p* < 0.001) reflects the cognitive impairment of the MCI subjects with a mean of 23.8/30 ± 2.7.

### General RT performance in the tasks

As regards the RT results acquired in the study, a 2 × 3 ANOVA was conducted between Group (MCI, HAA) and Task (SRT, CRT, and IMI). This analysis revealed a significant main effect for Task [*F*_(2.40)_ = 566.647, *p* < 0.001]. The RT was significantly greater in the IMI task than in the CRT task and was significantly greater in the CRT task than in the SRT task. In addition, this ANOVA showed a significant main effect for Group [*F*_(1.20)_ = 20.762, *p* < 0.001], which revealed a greater RT in MCI subjects than in their healthy counterparts.

**Figure 1:**
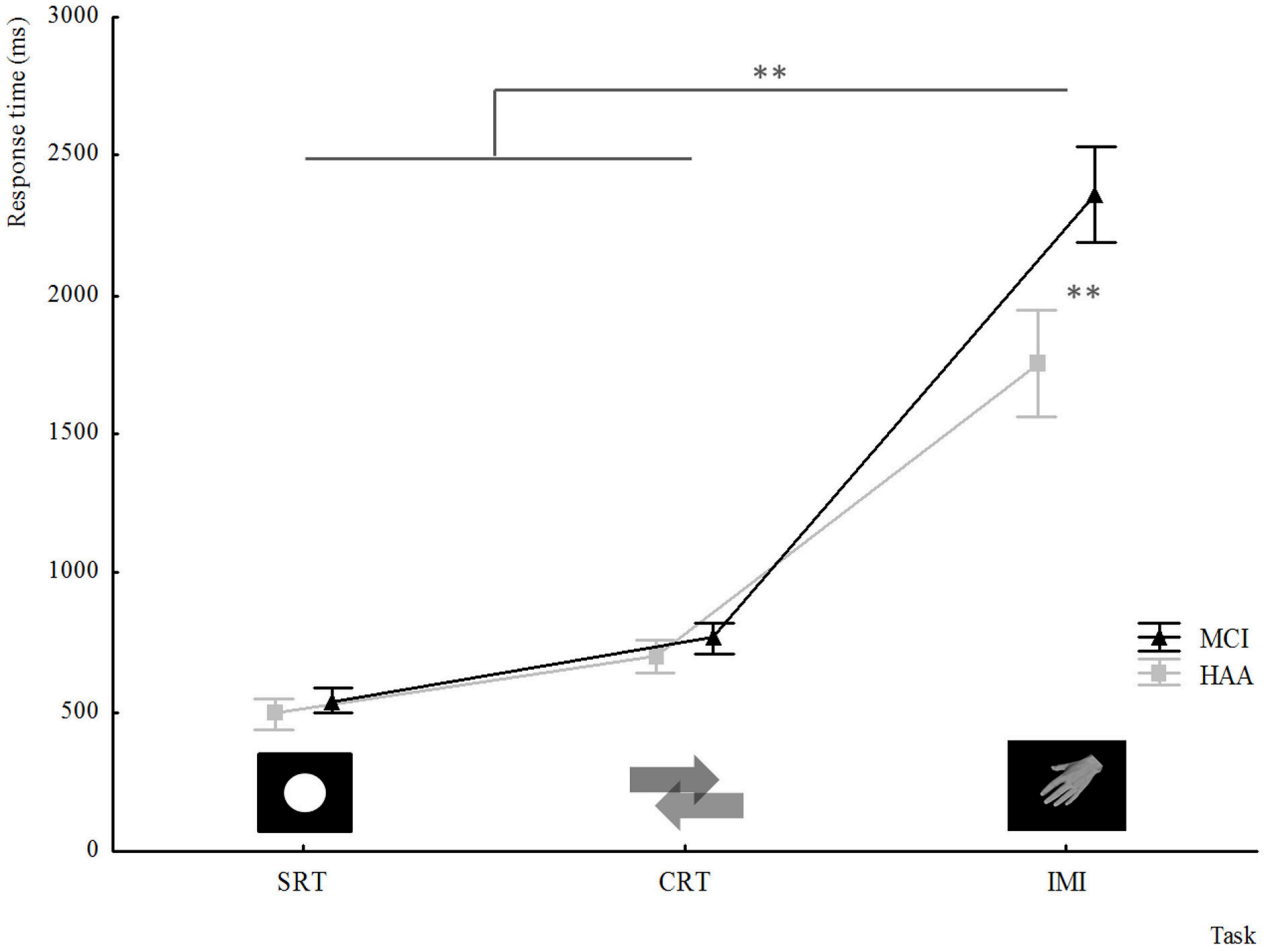
**Response time for different tasks collected in experiments for both groups HAA and MCI: SRT simple reaction time; CRT Choice reaction time; IMI implicit motor imagery**. ^**^indicates a significant difference *p* < 0.001.

Interestingly, there was an interaction between the Task and the Group [*F*_(2.40)_ = 20.116, *p* < 0.001]. The *post-hoc* analysis showed that MCI subjects were significantly slower than their healthy counterparts in the IMI task (*p* < 0.001; see Figure 1). Finally, there was no significant difference in RT between the two groups for SRT and CRT tasks.

### Analysis of RT performance in the IMI task

The ANOVA used to highlight the RT performance in the IMI condition was a three factor analysis with: Group; Orientation; Hand.

**Figure 2:**
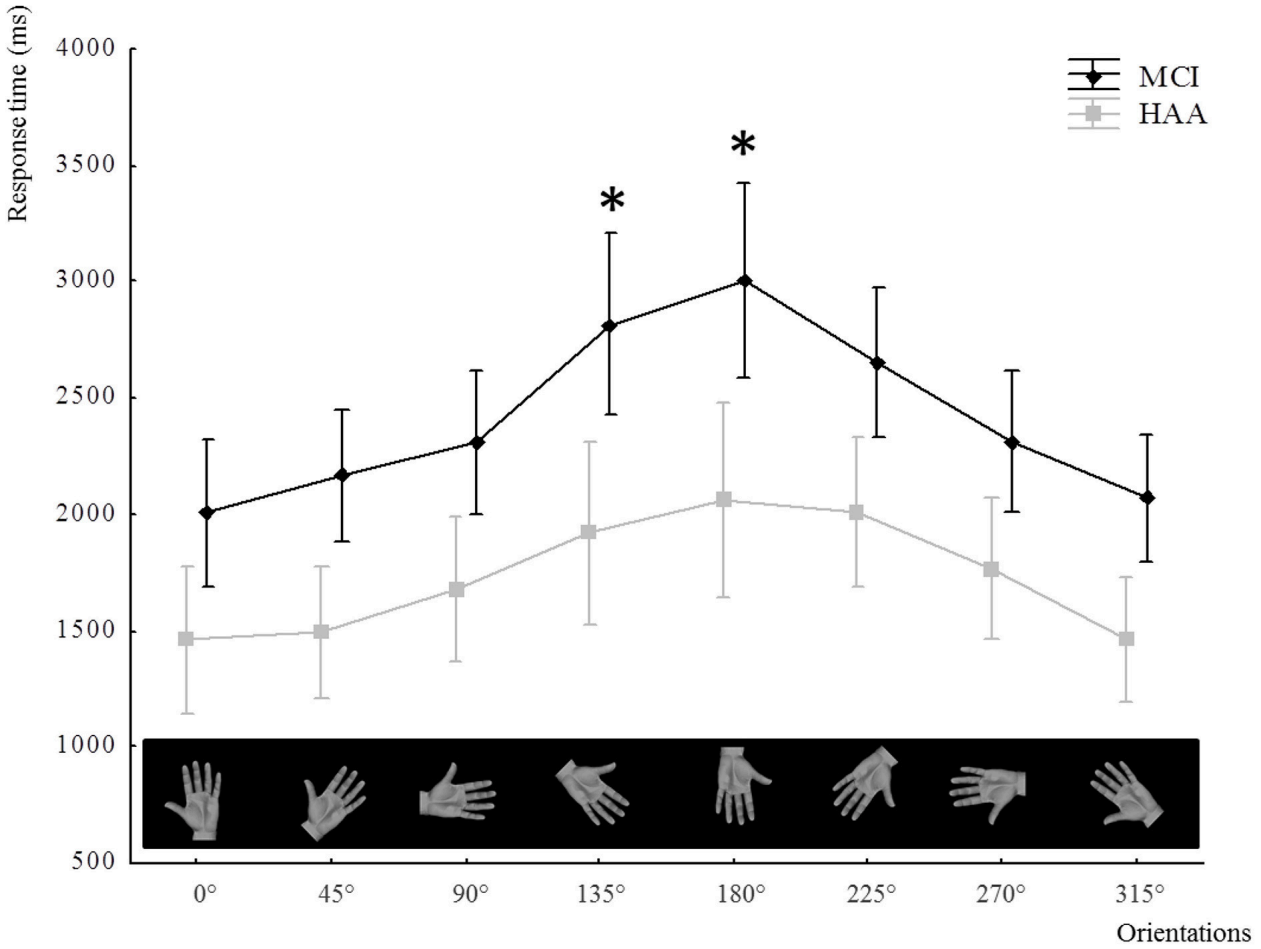
**The means of RT in the IMI task represented through the groups and all orientations**. The use of “^*^” signifies a significant difference in RT (*p* < 0.05) for one orientation compared with other orientations tested and a significant difference with RTs for healthy subjects for the same orientation. In the X-axis, the orientations of stimuli hand used in the study are illustrated only through the pictures of left hand, palm view.

As described above, this second analysis confirmed the significantly slower RT in MCI subjects than healthy subjects in the IMI task, with a significant main effect for Group [*F*_(1.22)_ = 29.383, *p* < 0.001]. Furthermore, the results showed a significant main effect for Orientation [*F*_(7.154)_ = 34.347, *p* < 0.001]. The *post-hoc* analysis showed that the furthest orientation of the hand stimuli from the reference position 0° (orientation: 135, 180, and 225°) increased the RT. Moreover, the analysis highlighted an interaction between Orientation and Group [*F*_(7.154)_ = 2.176, *p* = 0.03]. The *post-hoc* analysis showed that MCI participants were significantly slower than HAA subjects when performing mental rotation at angles of 135 and 180° (see Figure 2).

**Figure 3:**
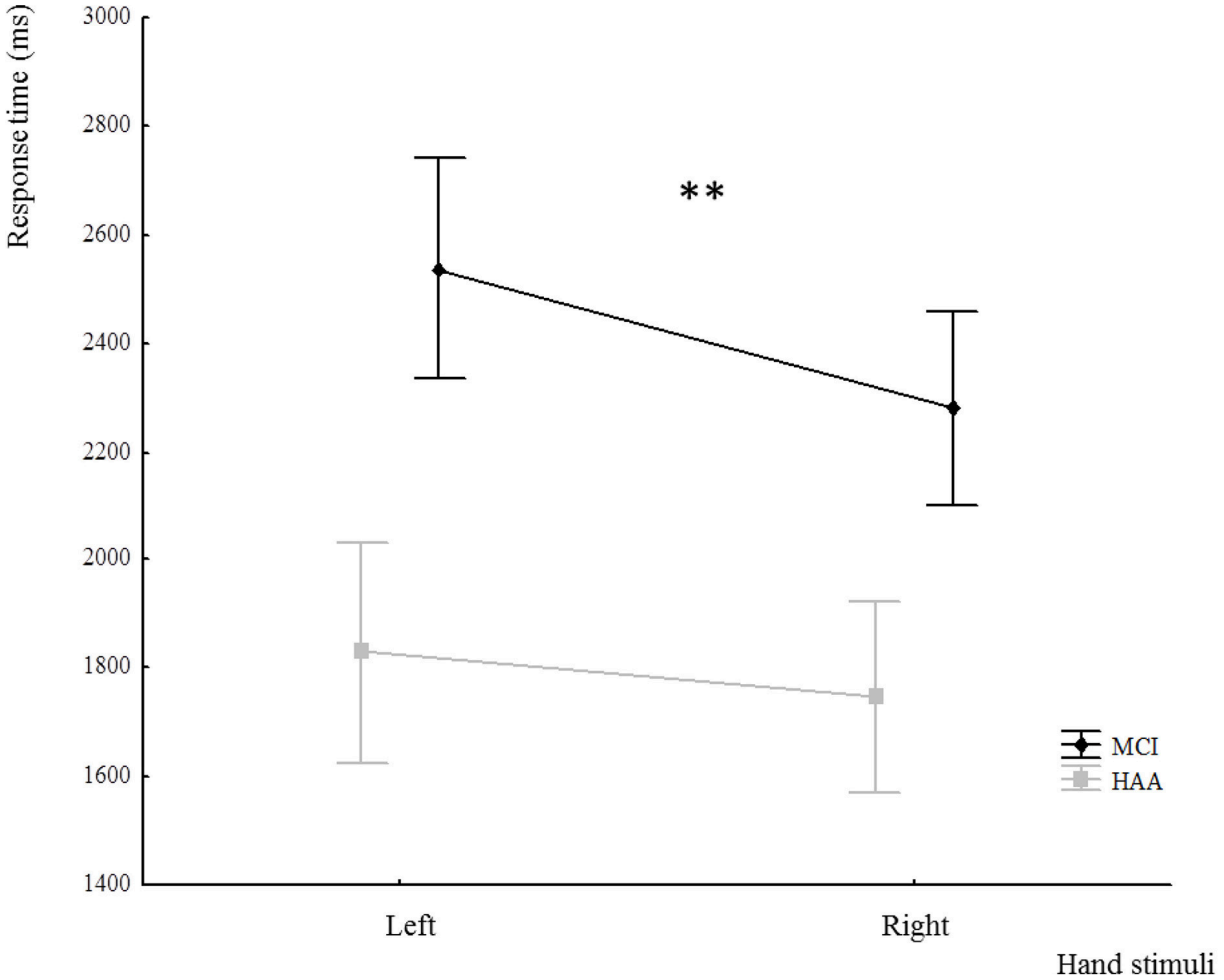
**Response time in the IMI task for both groups according to the hand stimuli used: left and right hand**. ^**^indicates a significant difference *p* < 0.001.

In addition, this analysis showed a significant main effect for Hand [*F*_(1.22)_ = 7.781, *p* = 0.01]. The participants were slower when the stimulus represented the non-dominant hand. Interestingly, there was an interaction between Hand and Group [*F*_(1.22)_ = 8.90, *p* < 0.001]. The *post-hoc* analysis revealed that the IMI task related to the non-dominant hand induced a significant increase in RT only in MCI subjects (*p* < 0.001) (see Figure 3).

### Error rate

The error rate assessed in the experiment concerned the CRT and IMI tasks. First, this analysis of the two tasks showed no main effect for Group but only a tendency [*F*_(1.22)_ = 4.043, *p* = 0.058] toward a greater error rate in the MCI group. We found a significant main effect for Task [*F*_(1.22)_ = 14.138, *p* = 0.001]. The participants had a greater proportion of wrong answer in the IMI task than in the CRT task. However, there was no significant interaction between Task and Group for “error rate” [*F*_(1.22)_ = 2.65, *p* = 0.117]. In both tasks, the number of wrong answers was similar in both groups.

The ANOVA used to highlight the error rate in the IMI condition was a three-factor analysis with: Group; Orientation; Hand. The results of the analysis showed a significant main effect for group [*F*_(1.22)_ = 6.118, *p* = 0.02]. Indeed, MCI subjects had a greater number of errors than their healthy counterparts. Finally, we noted a significant main effect for orientation [*F*_(7.154)_ = 21.710, *p* < 0.001] and no interaction between Orientation and Group [*F*_(1.154)_ = 0.473, *p* = 0.85].

## Discussion

The aim of the present study was to investigate the influence of amnestic MCI associated with AD on abilities in IMI, which concerns upper limb movement, through the task of hand laterality judgements (Parsons, 1987). The experimental design allowed us to assess the performance of MCI subjects and their healthy counterparts in IMI tasks.

In this study, the performance of MCI patients tested in both SRT and CRT tasks was comparable to that of their healthy counterparts. The SRT task implicates both sensory-motor and attentional processes (Storandt and Beaudreau, 2004), while the CRT task includes an additional decision-making process (Gordon and Carson, 1990). Concerning these tasks, our results showed no inter-group differences, which is in keeping with the literature (Levinoff et al., 2005; Makizako et al., 2013). In their study, Levinoff and colleagues showed no significant difference between MCI subjects and their healthy counterparts in either SRT or CRT, which contrasted sharply with the significantly longer RT in SRT and CRT tasks in patients with AD. In a wider context, the literature revealed differences in damage between MCI and AD patients with regard to cognitive and motor processes, with AD patients showing a significant decrease in attention and executive abilities (Petersen, 2004; Albert et al., 2011; Clément et al., 2013).

The analysis of IMI showed very interesting results. In both groups, reaction times were modulated by the orientation of the stimulus. These modulations of RT depending on the different orientations of the stimulus reflect the consideration of the angular distance required to execute the movement of transferring the hand from its initial toward its final position (Decety et al., 1989; Papaxanthis et al., 2003). First, we must point out that MCI subjects seemed to involve motor imagery processes to solve the task (IMI), because there their reaction times varied depending on the orientation of the stimulus. Second, MCI subjects showed slowing of this mental process (IMI) revealed by the longer reaction times for the combined stimulus orientations. Interestingly, this slowing down differed depending on the stimulus, and was significantly more pronounced for angles furthest from the initial position (i.e., 135 and 180°), considering the medial rotation needed to achieve the IMI task. In contrast, for lateral rotations needed to reach 225; 270; and 315°, the difference between groups was not significant. In an interesting EEG study, Ter Horst and colleagues showed that IMI processes are more involved in medial-mental rotations of the hand, as this task is in accordance with the biomechanical constraints of the overt execution of movement, than is the case in lateral-mental rotations, which probably involve a visual imagery process (Ter Horst et al., 2013). Our results support the notion that in MCI subjects the most challenging IMI processes are specifically impaired. As they took into account the orientation of the stimulus, but were impaired in the most challenging mental rotations, we strongly suggest that MCI patient suffer more from inaccurate IMI processes than an inability to perform this implicit representation of the action itself. Analysis of the error rate seems to support this deterioration in IMI processes in the MCI group by showing a trend toward a higher error rate in MCI subjects than in their healthy counterparts.

Interestingly, this influence of the angular distance as a predictor of the reaction time is also verified when considering the handedness of the stimulus. As shown in the results, the RT was longer in the MCI group for the non-dominant hand than for the dominant hand.

This interesting result could be interpreted according to two hypotheses. In the first, one may suppose that the relative under-use of the non-dominant hand is greater in individuals with cognitive decline. This could be supported by the literature on motor and functional impairment in the MCI population, which is even more widespread in the AD population (Gauthier et al., 2006; Albert et al., 2011). The rarefaction of movement could result in less frequent updating of the internal models of action, thus leading toward an increasing difficulty in the mental processes associated with these action representations (Wolpert and Ghahramani, 2000; Wolpert and Flanagan, 2001). Another hypothesis could be raised by considering the memory loss that characterizes the MCI population. As the non-dominant hand is less often used in everyday life, it is possible that it was more crucial to encode or recall the sensorimotor memory of this body region in the case of mental representation of an action involving this hand. Our result could also be explained by the impairment of these memory processes in amnestic MCI patients (Celone et al., 2006; Dickerson and Sperling, 2008).

## Conclusion

In the light of our results, we can suppose that MCI patients are able to engage in IMI processes, but still show substantial impairment of this mental ability across its complexity. This corresponds to modifications of motor representation, which could lead to the worrisome impairment of the movement itself over the course of the disease. This phenomenon is increasingly documented in the literature (Albers et al., 2014). To validate the second hypothesis, it would be very interesting to follow the MCI patients recruited in this study to determine whether or not individuals presenting the worst IMI abilities will be faced with the worst functional evolution.

### Conflict of interest statement

The authors declare that the research was conducted in the absence of any commercial or financial relationships that could be construed as a potential conflict of interest.
